# MDM2 SNP309 is associated with high grade node positive breast tumours and is in linkage disequilibrium with a novel MDM2 intron 1 polymorphism

**DOI:** 10.1186/1471-2407-8-281

**Published:** 2008-10-01

**Authors:** Fiona EM Paulin, Mary O'Neill, Gillian McGregor, Andrew Cassidy, Alison Ashfield, Clinton W Ali, Alastair J Munro, Lee Baker, Colin A Purdie, David P Lane, Alastair M Thompson

**Affiliations:** 1 Department of Surgery and Molecular Oncology, Ninewells Hospital and Medical School, Dundee, DD1 9SY, UK; 2 Department of Molecular & Cellular Pathology, Ninewells Hospital and Medical School, Dundee, DD1 9SY, UK; 3 Department of Oncology, Ninewells Hospital and Medical School, Dundee, DD1 9SY, UK; 4 National Translational Cancer Research Network, UK; 5 Breast Cancer Research UK, UK; 6 University of Dundee, Nethergate, Dundee, DD1 4HN, Scotland, UK; 7 National Health Service Tayside HQ, Kings Cross, Clepington Road, DUNDEE, DD3 8EA; 8 Cancer Research UK, UK

## Abstract

**Introduction:**

A functional polymorphism within MDM2, SNP309 T>G, has been linked to early onset cancer. This study examined clinical associations of breast cancer with SNP309 in a Scottish Caucasian population and investigated additional MDM2 intron 1 polymorphisms.

**Methods:**

Intron 1 of MDM2 was PCR amplified and directly sequenced from 299 breast cancer patients and 275 cancer free controls and compared with clinical and pathological parameters.

**Results:**

SNP309 was observed, for the control and breast cancer cohorts respectively, at frequencies of: T/T = 44.7% and 39.5%; G/T = 42.2% and 47.2%; G/G = 13.1% and 13.4%, indicating that SNP309 is not a predisposing factor for breast cancer. The 309G/G genotype was associated with high grade tumours (OR = 1.64, 95%CI = 1.06–2.53, p = 0.025) and greater nodal involvement (OR = 2.51, 95%CI = 1.26–4.98, p = 0.009). SNP309 was not associated with an earlier age of cancer diagnosis. No association was observed between genotype and age of breast cancer diagnosis when patients were stratified by menopausal status and estrogen receptor status. Three additional low frequency SNPs were identified: 344T>A, 285G>C and 443G>T, the latter two novel. SNP285 was in complete linkage disequilibrium with SNP309 (D' = 1.0) with the minor alleles being in phase with each other. Moreover, the 285C/C, 309G/G double homozygous genotype was only observed in the breast cancer cohort.

**Conclusion:**

SNP309G/G is associated with poor prognostic breast cancer features in the Scottish population. Additionally, a novel SNP, SNP285, that is in linkage disequilibrium with SNP309, may also have a role in breast tumorigenesis.

## Introduction

MDM2, encoded by the human homologue of Murine Double Minute oncogene, is the principal negative regulator of p53, a transcription factor which plays key roles in cell division and response to DNA damage [1,2]. p53 is frequently mutated in cancer resulting in defective functions, including apoptotic and cell cycle arrest programs [3]. MDM2 controls p53 levels and activity by a number of different mechanisms, including direct inhibition of the transcriptional activity of p53 [4]. In addition, MDM2 acts as an E3 ubiquitin ligase targeting p53 for nuclear export and proteosomal degradation [5]. Furthermore, as MDM2 is a transcriptional target for p53, through the P2 inducible promoter located in intron 1, a finely balanced negative feedback loop mechanism exists [6].

Development of cancer is often associated with defects in this p53-MDM2 regulatory circuit, and in cells with wild type p53 other alterations in the p53 pathway are often observed [3]. MDM2 is overexpressed in a number of different cancers and in breast cancer, where only 30% of tumours have mutated p53, some 40% display overexpression of MDM2, although amplifications are rare [7-10].

A single nucleotide polymorphism (SNP) within intron 1 of MDM2, a T to G substitution (T>G) at position 309 (SNP309) (rs2279744), has been shown to lead to enhanced binding of the Sp1 transcription factor resulting in elevated levels of both MDM2 mRNA and protein, thereby attenuating the p53 response [11]. In Li-Fraumeni patients, individuals homozygous or heterozygous for SNP309 (G/G or G/T) were shown to develop cancer at an earlier age than wild type individuals; in patients with sporadic soft tissue sarcomas, the 309G/G genotype correlated with an average 12 year earlier age of diagnosis [11]. SNP309 was thus postulated as a potential modulator of cancer susceptibility [11].

Subsequent studies of the SNP309 polymorphism have demonstrated variable frequencies of 309G/G depending on race and ethnicity [12-15]. Similarly, the association between SNP309 and development of cancer has produced conflicting data (reviewed in [16]). This study therefore sought to sequence the MDM2 intron 1 region around SNP309 in detail and determine SNP frequencies from a control cohort of Scottish Caucasians (n = 275) and a cohort of geographically matched Scottish Caucasian women with breast cancer (n = 299). The MDM2 SNP genotypes were examined to determine if they could be linked to an increased cancer susceptibility, age of cancer diagnosis, pathological variables and clinical outcome.

## Methods

### Patient & control samples

Venous blood samples were obtained from otherwise unselected consenting patients (299) with a diagnosis of primary breast cancer attending routine breast cancer clinics at Ninewells Hospital, Dundee between 1999 – 2005. Age at first cancer, menopausal status at diagnosis, family history of breast cancer, estrogen receptor (ER), progesterone receptor (PgR), HER-2 expression, pathological nodal status, tumour grade and Nottingham Prognostic Index (NPI) were recorded. Tumour grading was carried out by a specialist pathologist (CAP) and graded as defined by the NHS Breast Screening Programme guidelines [17]. NPI was calculated as described [18] and then classified into Poor (>5.4), Moderate (3.4–5.4) and Good (<3.4) prognosis. ER and PgR were scored according to the quickscore method [19] and a score ≥ 4/18 was considered to be positive. HER-2 was evaluated by immunohistochemistry and samples scoring 2+ by IHC were then subjected to fluorescent in situ hybridisation. Samples scoring IHC 3+ or IHC 2+/FISH +ve were considered to be HER-2 positive [20]. Controls (275) were not matched to cases, but were similar in age (Mean age = 53.0 yr, Range = 9.8 – 95 yr), had no prior history of cancer and were all of Caucasian descent residing in the Tayside region. All samples were collected with informed consent in compliance with all principles of the Helsinki Accord and approved by the Tayside Local Research Ethics Committee.

### Isolation of Genomic DNA

Genomic DNA was extracted from heparinised venous blood using the Qiagen BioRobot EZ1 (Qiagen), with standardised protocols (EZ1 DNA Blood Card) as recommended by the manufacturer (Qiagen). Samples were quantified by spectrophotomeric reading using the NanoDrop^® ^ND-1000 (NanoDrop Technologies) and stored at -80°C.

### PCR amplification and sequence analysis

50 μl PCR reactions were set up using a standard premix of; 10 × PCR buffer (Promega), 10 nmol each dNTP (Epicentre Biotechnologies) and 20 pmol primers (MWG Biotech AG): MDM2_SNP309F: ^5'^GCGGGAGTTCAGGGTAAAGG^3'^, MDM2_SNP309R: ^5'^CTCCAAT CGCCACTGAACAC^3'^. Reactions were hot started with 1 unit of Taq polymerase (Promega) and run with conditions of denaturation at 95°C for 2 min, followed by cycling at 95°C (30 s), 60°C (15 s), 72°C (30 s), with final elongation at 72°C (5 min). PCR was performed in an Applied Biosystems 9600 thermocycler with a heated lid. The resulting 287 bp products were then run on 3% w/v agarose gels (Genseive LE agarose) and visualised using a UV transilluminator. The PCR products generated were purified using a modification of the ExoSAP enzymatic clean-up method. 5 μl of PCR product was incubated with 1 U of Exonuclease I and 1 U of Shrimp Alkaline Phosphatase for 20 min at 37°C then inactivated by incubating at 80°C for 15 min. The purified products were directly sequenced using the ABI PRISM^® ^BigDye™ Terminators V 3.0 sequencing kit and run on an ABI 3130 genetic analyzer (Applied Biosystems).

### Cloning and Sequencing

Genomic DNA was PCR amplified as above and then ligated in the vector pGEM^®^-T Easy according to the manufacturer's instructions (Promega). *E. coli *JM109 cells were transformed, white colonies picked and then subjected to plasmid isolation and restriction analysis. Plasmids containing the correct insert were sequenced using the M13 Reverse Primer.

### Statistical analysis

The associations between genotypes and risk of breast cancer were calculated by determining the odds ratios (ORs) and 95% confidence intervals (CI) from logistic regression analyses. Associations between genotype and age of cancer diagnosis were determined using Fisher's exact test (2 sided) and 2-sample t-tests. Genotype frequencies and association with other clinical parameters were calculated from logistic regression analyses by determining the ORs and 95% CIs. Haplotype analysis was performed using Haploview software [21]. All analyses were performed using Microsoft Excel 2002 SP3 (Microsoft Corporation) and Minitab Release 14.13 (Minitab Inc.). The null hypothesis was rejected at an α level of 5% for all analyses.

## Results

### SNP309 frequencies in a control and breast cancer population

SNP309 frequencies were examined in a control cohort of Scottish Caucasian individuals (n = 275), consisting of both males (n = 93) and females (n = 182) (Table 1). SNP309 genotype frequencies were found to be consistent with the Hardy-Weinberg equilibrium and similar to those reported previously [22]. Minor differences observed between males and females were not statistically significant (p = 0.301, Fisher's exact test). SNP309 frequencies within the breast cancer population (n = 299) were: T/T (39.5%), G/T (47.2%) and G/G (13.4%). This distribution was similar to that of the female control population and logistic regression analysis revealed that no genotype was significantly associated with an increased risk of breast cancer (Table 1).

**Table 1:** Genotype frequencies of MDM2 SNP309 in Scottish Caucasian control and breast cancer populations

**309 SNP**	**Total**	**309T/T**	**309T/G**	**309G/G**
	n	n (%)	n (%)	n (%)
Control samples	275	123 (44.7)	116 (42.2)	36 (13.1)
Males	93	48 (51.6)	34 (36.6)	11 (11.8)
Females	182	78 (42.8)	79 (43.4)	25 (13.7)
Breast cancer samples	299	118 (39.5)	141 (47.2)	40 (13.4)
Breast cancer vs. Female controls OR (95% CI)		1.0 (referent)	1.18 (0.80 – 1.87)	1.06 (0.60 – 1.87)
p value		-	0.41	0.85

MDM2 SP309 genotype frequencies in Scottish Caucasian control and breast cancer populations. OR = Odds ratio, CI = Confidence interval.

### SNP309 and age of breast cancer diagnosis

**Figure 1:**
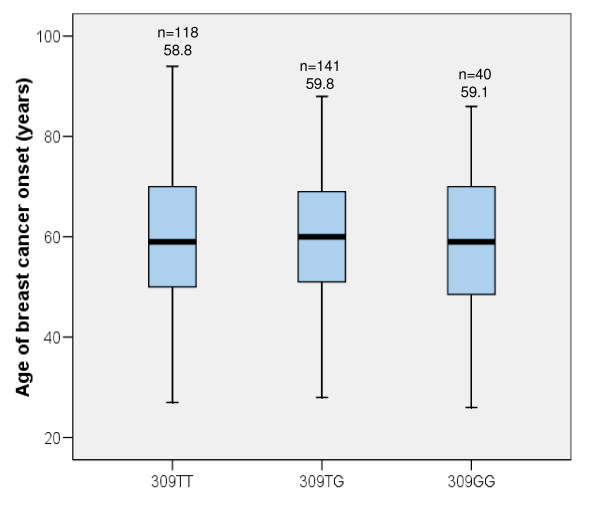
**Box and whiskers plot showing the age distribution of breast cancer diagnosis in the breast cancer population with respect to genotype.** Median values are represented by a solid horizontal bar, boxes denote the inter-quartile range and bars indicate the maximum and minimum values. Shown above is the mean value and number of samples per group. Overall mean = 59.3 years (n = 299).

To test if 309G/G was associated with an earlier age of cancer onset, as originally demonstrated [11], the age at first diagnosis of breast cancer was recorded and median values plotted with respect to genotype (Figure 1). No statistically significant differences were observed (mean age of diagnosis in years; T/T = 58.8, T/G = 59.8, G/G = 59.1) indicating that, in this population, SNP309 is not associated with age at diagnosis of breast cancer. 13 out of the 299 breast cancer patients had presented with other prior malignancies. However, the mean ages of any cancer diagnosis for each of the genotypes was not significantly altered, even when this was taken into consideration (data not shown).

### SNP309 and clinicopathological characteristics

We examined SNP309 genotype with respect to a number of clinicopathological features using logistic regression analysis (Table 2). 309G/G was significantly associated with higher tumour grade (OR = 1.64, 95%CI = 1.06–2.53, p = 0.025) and 309T/G associated with lower tumour grade (OR = 0.68, 95%CI = 0.53–0.88, p = 0.003). 309T/G was also associated with ductal carcinoma in situ (DCIS) but not observed with the 309G/G genotype (OR = 4.21, 95%CI = 1.67–10.44, p = 0.0012). The 309G/G genotype was also found to be significantly associated with node positive cancers (OR = 2.51, 95%CI = 1.26–4.98, p = 0.009). Corroborating evidence demonstrating that 309G/G was associated with these poorer prognostic markers was the association of 309G/G with an increased Nottingham Prognostic Index (NPI) (OR = 1.30, 95%CI = 1.02–1.65, p = 0.031), consistent with NPI values being a combinatorial score based on tumour grade, nodal status and tumour size.

**Table 2:** Association of MDM2 SNP309 genotype and clinicopathological characteristics.

	**309 SNP Genotype**	**Parameter category n (%)**				**OR (95% CI)**	**p**
All patients (n = 299)	T/T	118 (39.5)					
	T/G	141 (47.2)					
	G/G	40 (13.4)					

Menopausal status (n = 299)		Pre (n = 70)	Post (n = 229)				
	T/T	32 (45.7)	86 (37.6)			0.71 (0.42 – 1.23)	0.22
	T/G	28 (40.0)	113 (49.3)			1.46 (0.85 – 2.52)	0.17
	G/G	10 (14.3)	30 (13.1)			0.91 (0.42 – 1.96)	0.78
ER status (n = 299)		Positive (n = 226)	Negative (n = 71)				
	T/T	91 (40.3)	28 (39.4)			1.11 (0.643 – 1.94)	0.38
	T/G	108 (47.8)	31 (43.7)			1.12 (0.65 – 1.93)	0.67
	G/G	27 (11.9)	12 (16.9)			0.65 (0.31 – 1.35)	0.24
Tumour Grade (n = 298)		DCIS (n = 20)	1 (n = 47)	2 (n = 107)	3 (n = 124)		
	T/T	5 (25.0)	18 (38.3)	42 (39.2)	53 (42.7)	1.21 (0.93 – 1.56)	0.15
	T/G	15 (75.0)	25 (53.2)	53 (49.5)	48 (38.7)	0.68 (0.53 – 0.88)	**0.003**
	G/G	0	4 (8.5)	12 (11.2)	23 (18.5)	1.64 (1.06 – 2.53)	**0.025**
Nodal status (n = 298)		Positive (n = 128)	Negative (n = 170)				
	T/T	50 (39.1)	67 (39.4)			0.98 (0.62 – 1.58)	0.95
	T/G	53 (41.4)	88 (51.8)			0.66 (0.41 – 1.05)	0.07
	G/G	25 (19.5)	15 (8.8)			2.51 (1.26 – 4.98)	**0.009**
NPI (n = 297)		DCIS (n = 20)	Good (n = 72)	Moderate (n = 123)	Poor (n = 82)		
	T/T	5 (25.0)	30 (41.7)	47 (38.2)	35 (42.7)	1.00 (0.85 – 1.19)	0.95
	T/G	15 (75.0)	37 (51.4)	59 (48.0)	30 (36.6)	0.87 (0.73 – 1.03)	0.11
	G/G	0	5 (6.9)	17 (13.8)	17 (20.7)	1.30 (1.02 – 1.65)	**0.03**
HER-2 status (n = 196)		Positive (n = 47)	Negative (n = 149)				
	T/T	18 (38.3)	57 (38.3)			1.00 (0.51 – 1.97)	1.00
	T/G	19 (40.4)	73 (49.0)			0.71 (0.36 – 1.37)	0.30
	G/G	10 (21.3)	19 (12.8)			1.85 (0.79 – 4.31)	0.16
PgR Status (n = 294)		Positive (n = 181)	Negative (n = 113)				
	T/T	68 (37.6)	48 (42.5)			0.82 (0.49 – 1.36)	0.40
	T/G	90 (49.7)	49 (43.4)			1.29 (0.78 – 2.13)	0.29
	G/G	23 (12.7)	16 (14.1)			0.88 (0.42 – 1.88)	0.72

Association of MDM2 SNP309 genotype and clinicopathological characteristics. Each genotype was compared to all the other samples. OR = Odds ratio, CI = Confidence interval.

### SNP309 in relation to menopausal status and tumour ER status

Although no associations of SNP309 were observed between menopausal status or tumour estrogen receptor (ER) status the two parameters combined have been shown to be associated with 309 genotype [23]. This hypothesis is based on the fact that estrogen can regulate MDM2 levels, in part, via a promoter site adjacent to SNP309 and thus in pre-menopausal women, where there are higher levels of circulating estrogen, and in tumours that express high levels of the estrogen receptor, the effects of SNP309 may be exacerbated [24]. Indeed, in breast cancer patients, the 309G/G genotype was found more frequently in pre-menopausal patients expressing high tumour levels of estrogen receptor implying the importance of this SNP in breast cancer tumorigenesis [23].

**Figure 2:**
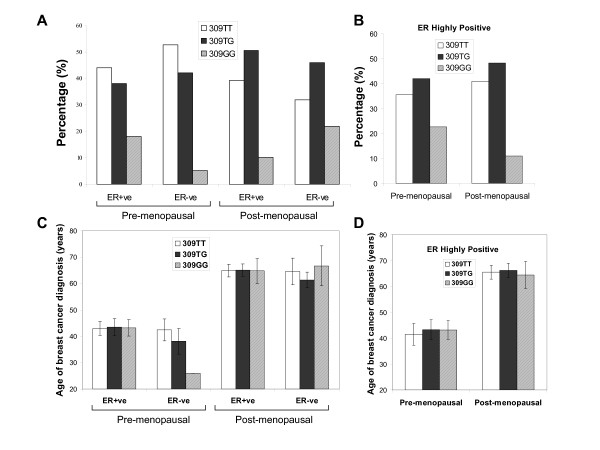
**SNP309 genotype distribution in relation to menopausal status and tumour estrogen receptor status.** A – ER positive defined as ER Score ≥ 4. ER negative defined as ER Score <4. B – Highly ER positive breast tumours defined as ER Score ≥ 12. Values expressed as a percentage for each group. The mean age of breast cancer diagnosis in relation to menopausal status and tumour estrogen receptor status. C – ER positive defined as ER Score ≥ 4. ER negative defined as ER Score <4. D – Highly ER positive breast tumours defined as ER Score ≥ 12. Error bars ± 95%CI.

To test if SNP309 was more common in ER positive patients compared to ER negative patients the menopausal status (as determined by factual statement) and ER status were then combined and examined. 50 out of 69 pre-menopausal patients (72%) and 176 out of 226 post-menopausal patients (78%) were ER positive (ER score ≥ 4). The 309G/G genotype was more frequent in the pre-menopausal ER positive patients (18.0%) compared with post-menopausal ER positive patients (10.2%) although non-significantly (Fisher's Exact test; p = 0.143) (Figure 2A). This effect was further heightened by subdividing the ER positive patients into a group with very high ER expression (ER score ≥ 12), more similar to the scoring applied by Bond *et al. *(Figure 2B) [23]. A higher relative frequency of the 309G/G genotype was observed in the pre-menopausal highly ER positive patients (22.5%) compared to the post-menopausal highly ER positive patients (10.9%), however, again this failed to achieve statistical significance (Fisher's Exact test, p = 0.135). Comparing the genotype distributions of ER positive/ER negative in the pre and post-menopausal patients, whether separated by ER score ≥ 4 or ≥ 12, suggests that estrogen may well be interacting with SNP309. No difference in the mean age of diagnosis between genotypes, in any of these groups, was observed (Figures 2C &2D).

### Additional MDM2 intron 1 polymorphisms

**Figure 3:**
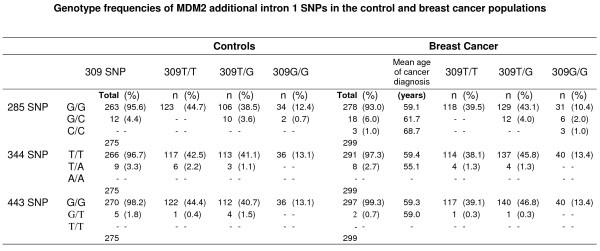
**Genotype frequencies of additional MDM2 intron 1 SNPs observed in the control (male & female) and breast cancer populations and their distribution with respect to SNP309 genotype.** Also shown is the mean age of breast cancer diagnosis for each group. Overall mean = 59.3 years (n = 299).

Upon sequencing MDM2 intron 1 three additional SNPs were detected in the control and breast cancer populations; 344T>A; 285G>C and 443G>T, the latter two representing previously unreported polymorphisms (Figure 3). SNP344 and SNP443 were found at very low frequencies; 344T/A heterozygotes (3.3%) and (2.7%), 443G/T heterozygotes (1.8%) and (0.7%) in the control and breast cancer cohorts respectively. Both these genotypes were in Hardy-Weinberg equilibrium and were found at similar frequencies within the control and breast cancer cohorts implying that neither is associated with breast cancer. SNP285 occurred at a slightly higher frequency; heterozygotes (4.4%) and (6.0%) in the control and breast cancer cohorts respectively. The homozygous 285C/C variant was observed only in the breast cancer cohort. In addition, in the breast cancer cohort, the minor allele frequency (285C) was higher (0.040) compared to the control (0.022) cohort (OR = 1.87, 95%CI = 0.93–3.78) and SNP285 appeared to deviate from Hardy-Weinberg equilibrium (Χ^2 ^= 14.29, p = 0.0001), however, numbers are too low to draw any firm conclusions. No genotype displayed statistically significant differences in mean age at breast cancer diagnosis.

Each of the intron 1 SNPs was then examined with respect to SNP309 (Figure 3). The 344T/A and 443G/T SNPs were both found in combination with the wild type 309 sequence (309T/T) and the heterozygote 309 sequence (309T/G) but not observed with the homozygous 309 sequence (309G/G). In contrast, 285G/C was found only with the heterozygous 309 sequence (309T/G) and the homozygous 309 SNP (309G/G). Moreover, individuals with the 285/309 double homozygote genotype (285C/C, 309G/G) were only observed in the breast cancer cohort (Chi-squared, p = 0.175).

The association between the 285G/C heterozygote, only observed with 309G/G and 309T/G but not 309T/T (in the control and breast cancer populations), and the homozygous 285C/C genotype, only observed with 309G/G (in the breast cancer population), suggests that the 285C and 309G alleles are in linkage disequilibrium. This was confirmed by carrying out haplotype analysis using Haploview software [21] (D' = 1.0) (r^2 ^= 0.047 and 0.071 for the control and breast cancer cohorts respectively) and indicates that the minor alleles are in phase with each other. This was further verified experimentally, by PCR amplification of intron 1 from ten individuals heterozygous for both loci (285G/C, 309T/G) cloning the products and then sequencing a minimum of eight colonies from each. In all cases, the 309G allele co-segregated with the 285C allele, whereas the 309T allele was always found on the same chromosome as the 285G allele. This suggests that 285C and 309T are mutually exclusive events indicating that the SNPs at positions 285 and 309 impact on each other potentially through chromosomal conformation or by binding accessory factors.

## Discussion

The intron 1 region of MDM2 was examined in a Scottish population of 299 breast cancer patients and 275 cancer free controls to establish any associations between MDM2 SNPs and breast cancer. In the breast cancer cohort MDM2 SNP309 was also analysed with respect to age at cancer diagnosis and pathological variables. The well characterised SNP309 and three additional SNPs: 344T>A, 285G>C and 443G>T, were identified in both the control and cancer populations. SNP 344T>A has been reported previously [11], but not studied in detail, and the latter two SNPs, to our knowledge, represent novel polymorphisms. SNP443G>T lies within the conserved 5'-RRRCWWGYYY [0,13]RRRCWWGYYY-3' p53 response element [6,25], implying that this SNP may alter the ability of p53 to bind and regulate the MDM2 intron 1 promoter, thereby influencing cancer susceptibility. However, a larger cohort would be required to test the clinical significance of this observation.

Neither the 344T>A, nor the 443G>T SNP were observed with the 309G/G genotype, probably due to the very low frequencies of each allele. In contrast, SNP285 G>C, which also has a low prevalence was always observed with the SNP 309T>G, either in the heterozygous (309T/G) or homozygous (309G/G) states, but never observed with the commonest 309T/T allele. This implies that 309SNP and 285SNP are in linkage disequilibrium and was confirmed by haplotype analysis. SNP 285 and SNP309 thus may influence each other and together modulate the levels of MDM2 produced. Supporting this potential role of SNP285 enhancing the effects of 309G/G, we observed the double homozygous genotype (285C/C, 309G/G) only in the breast cancer cohort and the heterozygous/homozygous genotype (285G/C, 309G/G) was more prevalent in the breast cancer cohort. Moreover, SNP285 appeared to deviate from Hardy-Weinberg equilibrium only in the breast cancer cohort. However, although potentially interesting, the numbers in our study are exceptionally small and therefore to verify if the combination of 309G/G together with the 285C variant may be breast cancer associated, a much greater number of patients would be required.

The comparable frequencies of T/T, G/T and G/G SNP309 for the control and breast cancer cohorts suggests that the SNP309G/G genotype did not significantly influence breast cancer occurrence within this Scottish population, consistent with studies of both sporadic and familial breast cancer [15,16,21,26-34]. Similarly, no increased cancer risk, associated with the 309G/G genotype, has been observed for other cancer sites including: colorectal cancer [35-37], uterine leiomyosarcomas [36], squamous cell carcinoma of the head and neck [36] and lung cancer [38]. In contrast, several studies, encompassing a diverse array of other tumour types but also including colorectal and lung cancer, have reported an increased risk of cancer occurrence associated with 309G/G [14,39-43]. While the frequencies observed in our studies are similar to those reported in other Caucasian populations, SNP309 genotypic frequencies do vary considerably between different ethnic groups [15,31]. For example, approximately 3% of African Americans carry the double homozygous 309G/G locus compared to frequencies of approximately 30% in the Japanese and Korean populations [12,13,15,31]. This may, in part, explain the discrepant associations between the 309G/G genotype and cancer. Additionally, from our work, other SNPs e.g. SNP285, which may also display variations in demographic frequencies, may influence the penetrance of SNP309. Furthermore, the higher frequency G allele in females confirms that appropriate controls should be used when interpreting results.

We did not observe an association between 309SNP and earlier age of cancer diagnosis unlike previous investigations [11,23,33], although the majority of studies in breast cancer are consistent with our findings [16,26-28,31,32,34]. Further studies by Bond *et al.*, 2006, showed that accelerated age of onset in 309G/G patients was observed only in females and, in diffuse large B-cell lymphomas and soft tissue sarcomas the 309G/G genotype had a higher incidence in pre-menopausal compared to post-menopausal patients. In our breast cancer cohort, there was no difference in SNP309 frequencies in pre- and post-menopausal patients. In the same study Bond *et al. *also examined invasive breast cancer stratified by ER status in addition to menopausal status and demonstrated both an increased frequency of 309G/G and an earlier age of onset associated with ER positive tumours in pre-menopausal women. In the present series, a higher frequency of the 309G/G genotype was observed in the pre-menopausal ER positive patients (ER ≥ 4) compared to the post-menopausal ER positive patients which was further enhanced by selecting a highly ER positive group (ER ≥ 12). Nonetheless, within any sub-classification no differences in the mean age of breast cancer diagnosis and genotype were seen.

These findings, demonstrating a higher proportion of 309G/G patients in the ER highly positive pre-menopausal group, are in agreement with Bond *et al., *2006 [23]. However, the proportions of pre- and post-menopausal highly ER positive tumours in the present study are very different to those reported previously: the highly ER positive (ER ≥ 12) pre-menopausal group had a frequency of 13.7% and the post-menopausal group had a frequency of 86.3%. In the two breast cancer cohorts studied by Bond *et al.*, the Caucasian Ashkenazi-Jewish group had values of 35.4% and 64.6% for the pre- and post-menopausal highly ER positive groups respectively, and values of 50.4% (Pre) and 49.6% (Post) in the second Caucasian population. This suggests that the demographics of the Scottish Caucasian population are very different to the American Caucasian population, which also appear to differ based on ethnicity. This may also explain, in part, why we did not observe an association with the mean age of cancer diagnosis. It also suggests that estrogen may not be the only variable that impacts on the penetrance of SNP309.

Compared with clinical and pathological features, the 309G/G genotype appeared to be associated with higher tumour grade and node positive cancers. Combining these parameters together with tumour size, to calculate NPI values, it was therefore not unexpected that 309G/G was linked with an elevated NPI score. From this preliminary study, the 309G/G genotype in the Scottish breast cancer population therefore appears to correlate with poor prognostic indicators, although a much larger cohort would be required to verify these findings. This association of 309G/G with higher grade tumours and increased nodal involvement is supported by a recent study in nasopharyngeal carcinoma where the 309G/G genotype was associated with advanced lymph node metastasis [39]. Other corroborating evidence comes from the observation that over-expression of MDM2 in cancer has been linked with increased levels of metastasis, poorer response to therapy and bad prognosis [44]. *In vitro *studies, utilising cell lines, have also shown that cells gain enhanced metastatic potential when MDM2 is up-regulated during hypoxic conditions [45].

Our data, taken together with that of the literature, suggests that the 309 genotype may be influencing the grade and metastatic potential of breast tumours. However, the presence of the G allele does not appear to be adding to this alone as the 309G/T genotype was found to be associated with low grade tumours. One potential explanation for this is that each of the different alleles could be binding different transcription factors. Indeed, the P2 promoter of MDM2 is known to be regulated by a myriad of different transcription factors including Sp1, p53, Ap1-ETS and MYCN [6,46-48]. Levels of these regulatory proteins and whether they are mutant or wild type, modified or unmodified, could also impact on MDM2 transcription levels. One key regulator influencing the penetrance of SNP309 may be p53, particularly as it is known to be haploinsufficient [49]. p53 has been examined in terms of the p53 polymorphism Arg72Pro [13,29,32,37,50] and mutational status of p53 [26,41,51-54] with conflicting results. Similarly, SNP285G>C that is in linkage disequilibrium with SNP309, identified in this study, may play a modulatory role influencing SNP309 penetrance. Thus, although all breast cancers can be classified on a histological and pathological level, this may in fact be determined by underlying SNP differences, some of which have greater influence than others depending on the population and the additional parameters examined.

## Conclusion

In conclusion, within this Scottish population the MDM2 309 polymorphism was found to associate with high grade cancers with greater nodal involvement and by implication poorer prognosis, but had no impact on the age of diagnosis of breast cancer. Furthermore, SNP309 is in linkage disequilibrium with SNP285 which may further modulate the penetrance of polymorphism effects.

## Abbreviations

SNP: single nucleotide polymorphism; DCIS: ductal carcinoma in situ; NPI: Nottingham prognostic index; ER: estrogen receptor; CI: confidence interval; OR: odds ratio

## Competing interests

The authors declare that they have no competing interests.

## Authors' contributions

MON carried out the PCR reactions and cloning. AC and GM carried out the sequencing reactions. AA and CA retrieved the clinical data. AJM and LB carried out the statistical analyses. CAP carried out pathological services. FEMP co-ordinated data collection, assimilated and analysed the data and prepared the manuscript. AMT and DPL conceived of the study, obtained financial support, managed the project and revised the draft critically. All authors read and approved the final manuscript.

## Funding

^§^National Translational Cancer Research Network, ^‡^Breast Cancer Research Scotland, ^#^University of Dundee, ^†^National Health Service Tayside, *Cancer Research UK.

## Pre-publication history

The pre-publication history for this paper can be accessed here:


